# Supplementary figures and images for: Flexible Resistive Gas Sensor Based on Molybdenum Disulfide-Modified Polypyrrole for Trace NO2 Detection
Source: Polymers (Basel). 2024 Jul 7;16(13):1940. doi: 10.3390/polym16131940 (PMC11244088; doi:10.3390/polym16131940)

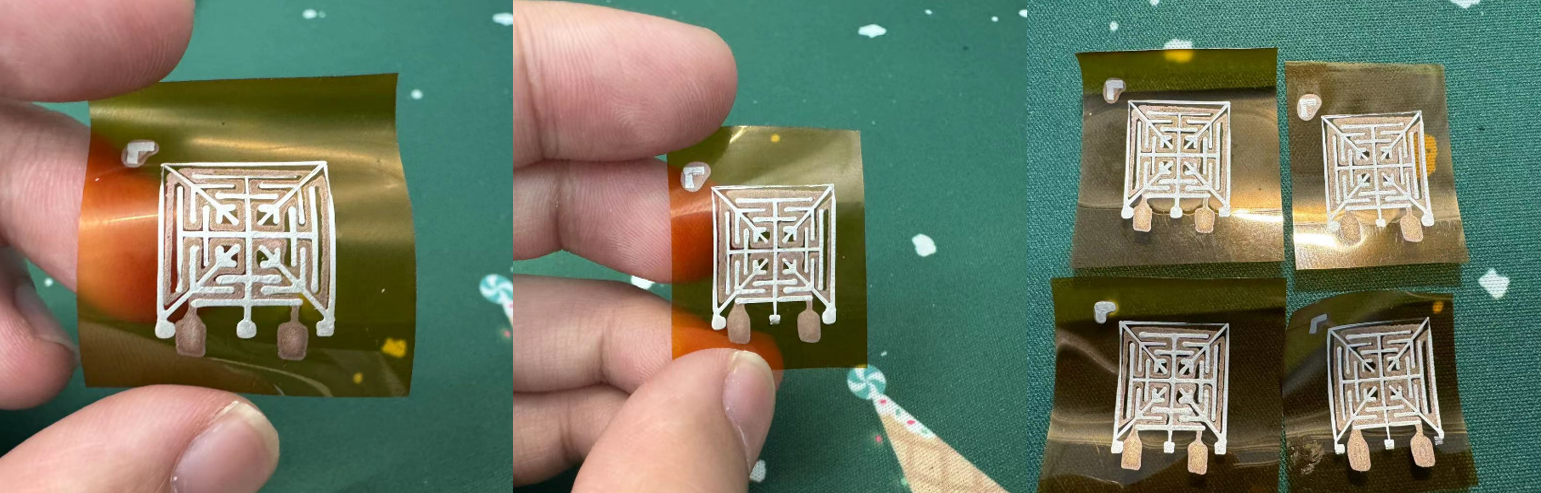

Supplement: Supplementary file 1 [file polymers-16-01940-s001.zip › Supporting Figure S1.tif]

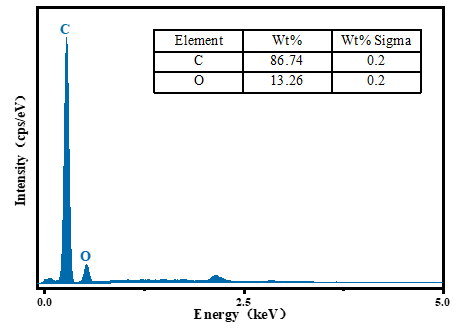

Supplement: Supplementary file 1 [file polymers-16-01940-s001.zip › Supporting Figure S2.tif]

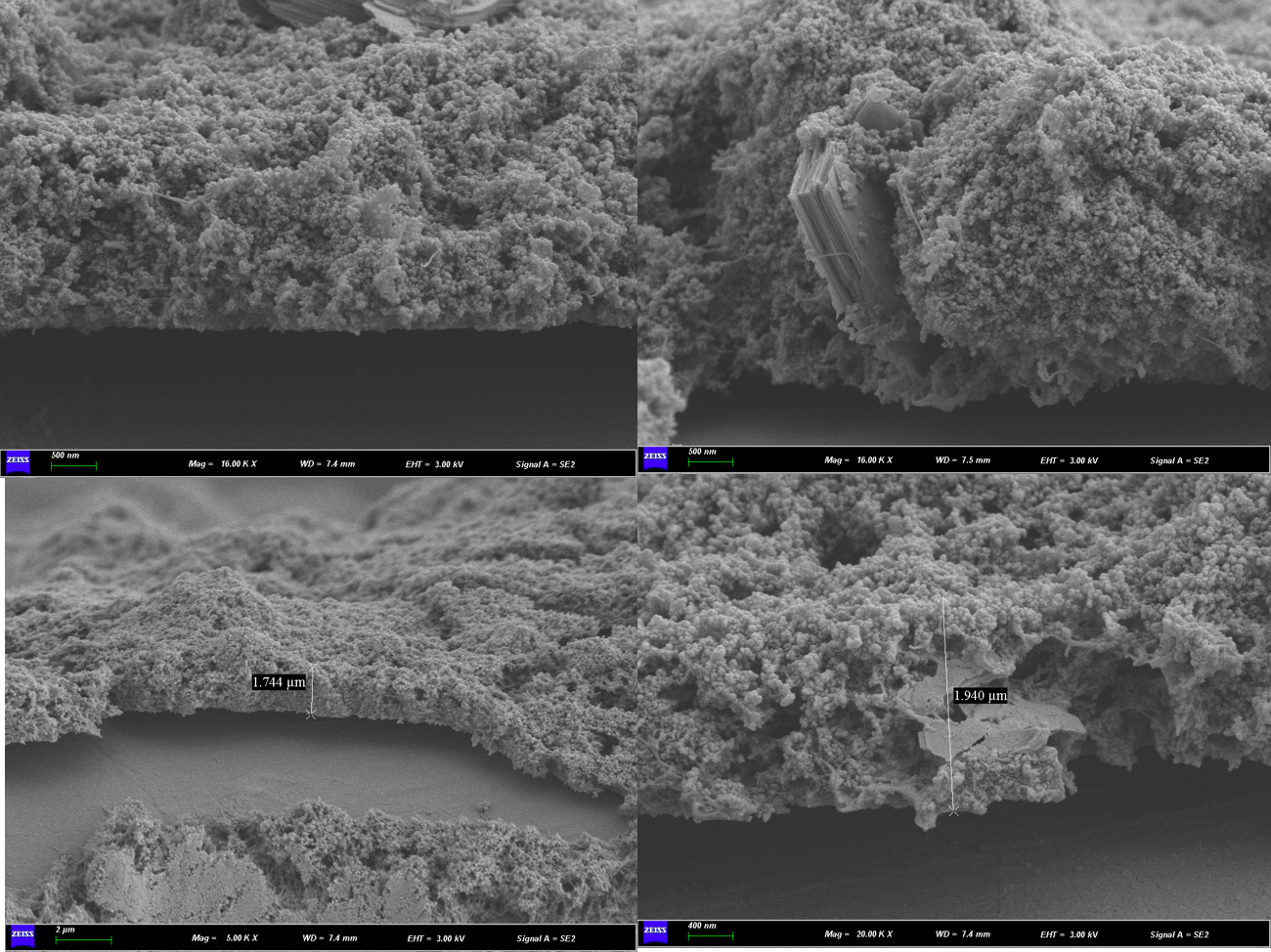

Supplement: Supplementary file 1 [file polymers-16-01940-s001.zip › Supporting Figure S3.tif]

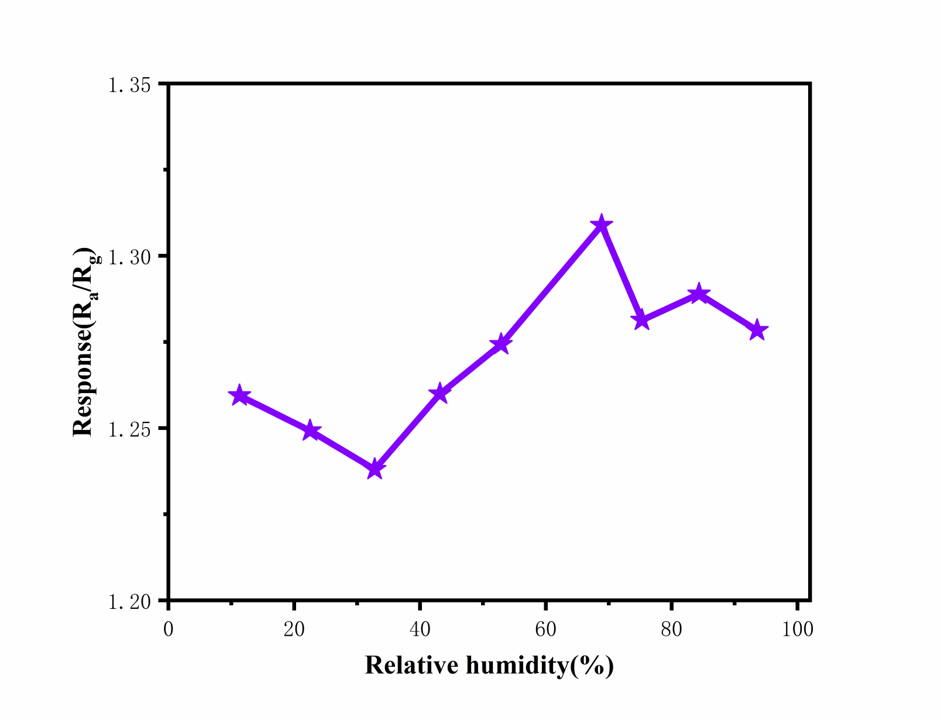

Supplement: Supplementary file 1 [file polymers-16-01940-s001.zip › Supporting Figure S4ú¿humidityú⌐.tif]

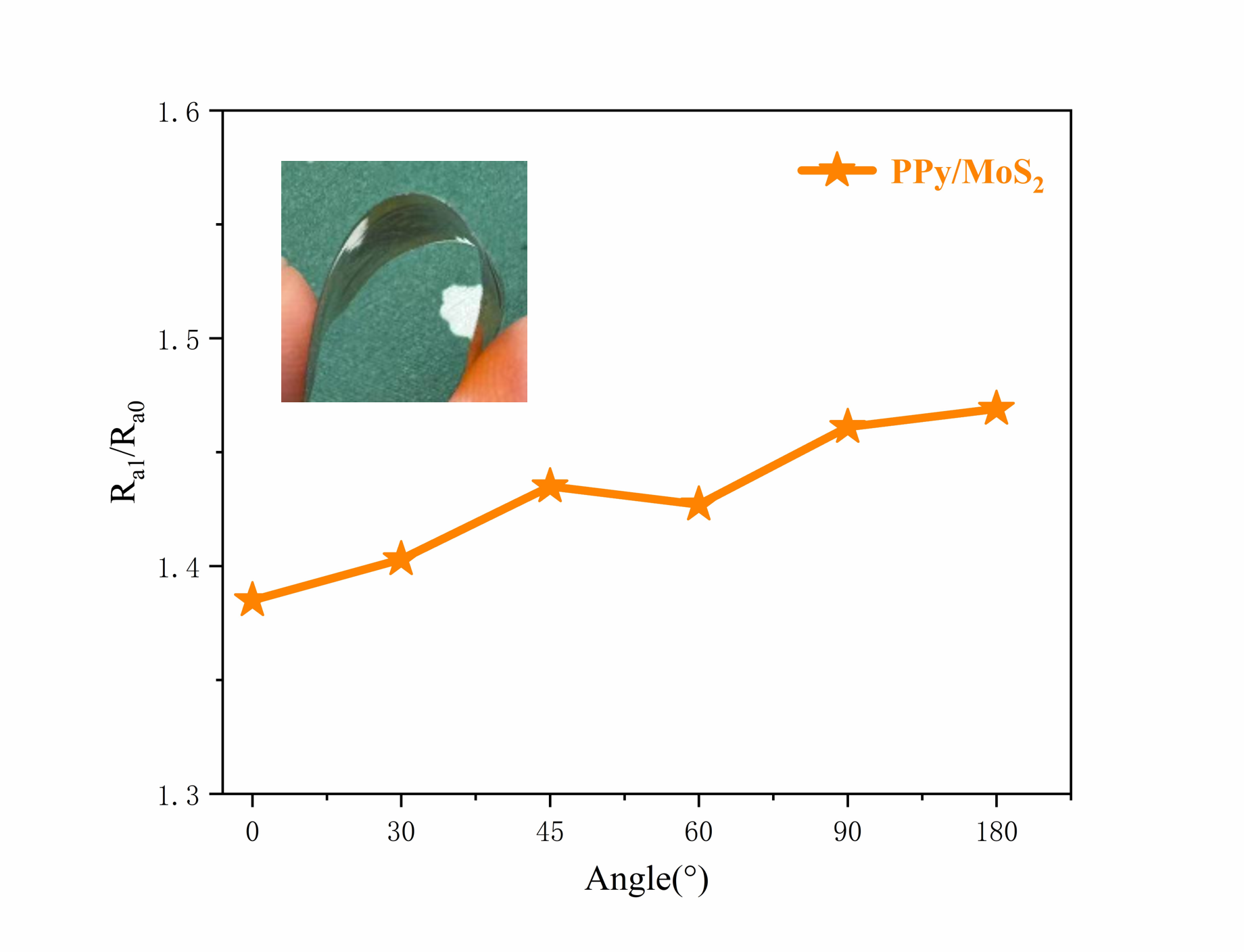

Supplement: Supplementary file 1 [file polymers-16-01940-s001.zip › Supporting Figure S5ú¿Angleú⌐.tif]
